# Effect of scheduled monitoring of liver function during anti-Tuberculosis treatment in a retrospective cohort in China

**DOI:** 10.1186/1471-2458-12-454

**Published:** 2012-06-19

**Authors:** Shanshan Wu, Yinyin Xia, Xiaozhen Lv, Yuan Zhang, Shaowen Tang, Zhirong Yang, Dehua Tu, Peiyuan Deng, Shiming Cheng, Xiaomeng Wang, Yanli Yuan, Feiying Liu, Daiyu Hu, Siyan Zhan

**Affiliations:** 1 Department of Epidemiology and Bio-statistics, School of Public Health, Peking University Health Science Centre, 38 Xueyuan Road Haidian District, Beijing, 100191, China; 2 Center for Tuberculosis Control and Prevention, Chinese Center for Disease Control and Prevention, Beijing, China; 3 Beijing Institute for Tuberculosis Control, Beijing, China; 4 Center for Drug Reassessment, State Food and Drug Administration, Beijing, China; 5 Center for Disease Control and Prevention in Zhejiang Province, Hangzhou, China; 6 Center for Disease Control and Prevention in Jilin Province, Changchun, China; 7 Center for Disease Control and Prevention in Guangxi Zhuang Autonomous Region, Nanning, China; 8 Center for Disease Control and Prevention in Chongqing Municipality, Chongqing, China

**Keywords:** Tuberculosis, Hepatotoxicity, Monitoring

## Abstract

**Background:**

Data on effect of regular liver function monitoring during anti-TB treatment is limited in China. This study aimed to evaluate the effects of scheduled liver function monitoring on identification of asymptomatic liver damage and anti-TB treatment outcomes during anti-TB treatment.

**Methods:**

A retrospective analysis was performed based on a national-level cohort study. A total of 273 patients developing liver dysfunction were divided into two groups, 111 patients who were diagnosed through scheduled liver function test within two months after initiation of anti-TB treatment formed scheduled monitoring group, others who were diagnosed due to developing symptoms formed passive detection group (n = 162). The two groups were compared through clinical features, prognosis of liver dysfunction and impact on anti-TB treatment using propensity score weighting analysis.

**Results:**

33.3% of 273 patients did not have any clinical symptoms, including 8 with severe hepatotoxicity. 1.8% in scheduled monitoring group and 11.1% in passive detection group required hospitalization (P = 0.004). Regarding the prognosis of liver dysfunction, most patients recovered, no death happened in scheduled monitoring group while 3 died in passive detection group. In terms of impact on anti-TB treatment, 35.1% in scheduled monitoring group and 56.8% in passive detection group changed their anti-TB treatment (P = 0.001).

**Conclusions:**

Scheduled monitoring is effective in identifying asymptomatic liver damage, reducing hospitalization rate and improving compliance of anti-TB treatment.

## Background

Tuberculosis (TB) is one of the most common infectious diseases worldwide, especially in developing countries. According to WHO reports, there were an estimated 8.5-9.2 million incident cases and 1.2-1.5 million deaths (including deaths from TB among HIV-positive people) in 2010[1]. As the second highest TB burden country in the world, China accounted for an estimated 12% of all TB cases worldwide [1]. China national surveys found a prevalence rate of bacteriologically-confirmed pulmonary TB of 119 (113–135) per 100, 000 population aged ≥15 years and six percent of TB patients died in 2010[1]. Standard short-course chemotherapy regimen, which comprises of a combination of Isoniazid(H), Rifampicin(R), Pyrazinamide(Z), Ethambutol(E) and Streptomycin(S) for 6–9 months is recommended by WHO and currently used in most high TB burden countries including China[2]. Due to the long duration of therapy and concurrent use of multiple drugs, adverse effects are regarded as the most important clinical consideration in patients undergoing anti-TB treatment [3]. Hepatotoxicity is the most serious one, which not only leads to high morbidity and mortality, but also diminishes anti-TB treatment effectiveness owing to non-adherence [4-7].

Several guidelines of anti-tuberculosis drug induced liver injury (ATLI) have been published and updated by the American Thoracic Society (ATS), the British Thoracic Society (BTS) and the Task Force of the European Respiratory Society, the WHO and the International Union Against Tuberculosis and Lung Disease [8-11]. Most of these guidelines suggest the need of regular liver function monitoring for preventing or alleviating ATLI, but all the recommendations are based on expert opinion and/or clinical experience of arbitrary authorities, which has not been rigorously tested[8,12]. Besides, there is no consensus on the frequency of monitoring. For example, the ATS recommends monitoring every 2 to 4 weeks for patients with possible risk for hepatotoxicity, whereas BTS recommends monitoring every week for the first 2 weeks and every fortnight for another 2 months [9,10]. In contrast, China has not published the relevant guideline of ATLI until now, and there is no explicit suggestion on monitoring for TB patients in China. No relevant study on the effect of regular monitoring of liver function has been published.

To address the issue of the effect of regular monitoring of transaminase during TB treatment, we conducted this retrospective analysis based on a national-level cohort study entitled ‘Anti-tuberculosis Drugs induced Adverse Reactions in China National Tuberculosis Prevention and Control Scheme Study (ADACS)’ [13,14]. Since the ADACS study was not specially designed to evaluate the effect of regular monitoring, only two routine tests (baseline and within 2 months) were conducted, we described “regular monitoring” as “scheduled monitoring” in this study. The findings will provide evidence and suggestion for developing management guidelines of ATLI in China.

## Methods

### Source population

**Figure 1:**
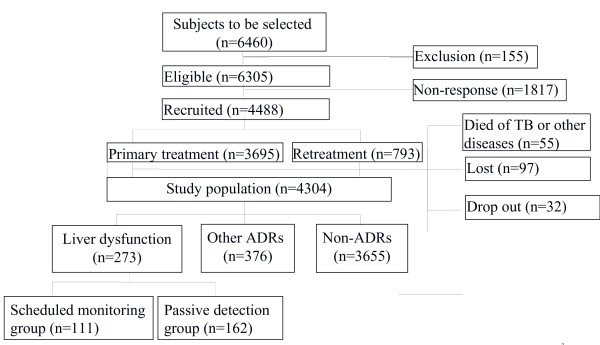
Flow chart of patients in ADACS.

Data were obtained from ADACS, which was approved by the Ethics Committee of Center for Tuberculosis Control and Prevention of China and carried out from October 2007 to March 2009[13,14]. And written informed consent was obtained from every participant or surrogate before enrolment. ADACS was a prospective longitudinal study of anti-TB drugs induced adverse effects, which consisted of a multi-center cohort of 4488 pulmonary TB patients and receiving standard short-course chemotherapy (primary and re-treatment patients would take HRZE/HRZES for initial intensive phase and HR/HRE for consolidation phase respectively) in four geographically and economically diverse areas of China[13,14]. During the study, patients were asked to take liver function test at baseline and within 2 months after treatment initiation in addition to developing unbearable symptoms [13,14], and for patients with abnormal baseline liver function the anti-TB treatment would not be started until their liver function turned normal through liver protective treatment, so the baseline liver function was normal for all patients. After the 6–9 months follow up period, 273 patients appeared liver dysfunction and 106 patients were diagnosed as ATLI according to the ATS criteria [14]. Of the 273 patients with liver dysfunction, 111 patients who were diagnosed through scheduled liver function test within two months after initiation of anti-TB treatment formed scheduled monitoring group, others who were diagnosed due to developing symptoms formed passive detection group (n = 162) (Figure 1). In this study, we compared the clinical features, prognosis of liver dysfunction and impact on anti-TB treatment between the two groups to determine the effect of liver function monitoring on identification of asymptomatic liver damage and anti-TB treatment outcomes during anti-TB treatment. Besides, all patients were required to fill in their ADACS calendars and record their everyday feelings as well as drug usages during the whole follow up period with the same pattern of treatment supervision [13].

### Assessment of liver dysfunction

Liver dysfunction was diagnosed as an increase in serum alanine aminotransaminase (ALT) or aspartate transaminase (AST) or total bilirubin (TBil) that was greater than the upper limit of normal (ULN), when other causes were excluded: new viral hepatitis infections, other liver diseases, other potentially hepatotoxic medications that would confound the picture. All assessments were double checked by two experienced physicians. The severity of liver dysfunction was classified according to the WHO Toxicity Classification Standards [12]: mild (ALT/AST ≤ 3 times ULN, or TBil ≤ 2 times ULN), moderate (3 times ULN < ALT/AST ≤ 5 times ULN, or 2 times ULN < TBil ≤ 5 times ULN) and severe (ALT/AST/TBil > 5 times ULN).

During the follow-up in ADACS, when liver dysfunction developed, patients would be given intensive monitoring and/or treatment (such as liver protective drugs or even hospitalization) according to the doctor’s clinical adjudication. The anti-TB treatment would not be changed immediately, only when ALT/AST/TBil elevated to greater than 2 times ULN, changes of anti-TB treatment would be considered by the doctor, including drug replacement, interruption, discontinuation, dose decrease and changes of medication administration.

### Outcome measures

For comparison of the two groups, the following parameters were noted: 1) the time period for the development of liver dysfunction from initiation of anti-TB treatment (liver dysfunction finding time); 2) requirement of hospitalization; 3) severity of liver dysfunction; 4) prognosis of liver dysfunction, which was defined and classified as recovered (ALT/AST/TBil turned normal), continuing with abnormal ALT/AST/TBil and death; 5) impact on anti-TB treatment.

### Statistical analysis

The baseline characteristics and clinical features of participants were described as median (inter-quartile range, IQR) for continuous variables (not subject to normal distribution), and percentages for categorical variables. Non-parameter test was used for continuous variables (not subject to normal distribution) and *χ*2 test was used for categorical variables.

In order to control the confounding bias in this observational study, propensity score weighting was used to compare the prognosis of liver dysfunction and impact on anti-TB treatment between participants in scheduled monitoring group and passive detection group. A propensity score model was developed with logistic regression model to predict patients’ assignment in this cohort. Covariate variables included in the model were as follows according to the selection principle [15]: age, gender, weight, marriage status, income per year, type of anti-TB treatment, history of anti-TB adverse reactions, history of liver diseases, HBSAg, pre-existing disease, pattern of taking anti-TB drugs (every day or every other day), and type of preventive liver protective drugs [12,16]. Liver protective drugs were classified as four categories (herbal preparations, finished manufactured herbal products, combinations of vitamins and other non-herbal substances, and pharmaceutical preparations) according to a modified version of WHO standard definitions for the evaluation and research of herbal medicines [17,18]. After the calculation of propensity score (PS), standardized mortality ratio weighting (SMRW) was used to adjust the data with formulas as follows:

(1) $$\text{Scheduled monitoring group}:{\text{W}}_{\text{a}}=1;\text{Passive detection group}:{\text{W}}_{\text{b}}=\left[\text{PS}\left(1-{\text{P}}_{\text{a}}\right)\right]/[(1-\text{PS}){\text{ P}}_{\text{a}}];{\text{P}}_{\text{a}}\text{is the percentage of scheduled monitoring group in this cohort}\text{.}$$

Then *χ*2 test was used to compare the prognosis of liver dysfunction and impact on anti-TB treatment between the two groups.

The statistical analyses were performed using SPSS for Windows (version 13.0; SPSS Inc.). A two-sided P value less than 0.05 was set as the significant level.

## Results

### Baseline characteristics

Of the 273 patients with liver dysfunction, 111 patients were in scheduled monitoring group while 162 patients were in passive detection group. The baseline characteristics of 273 patients were shown in Table 1. The median age of 273 patients was 40.0 years (IQR: 28.0-53.0), and 212 (77.7%) were male. Among the demographic features and weight, there was no significant difference between the two study groups. And no statistically significant difference was found in type of anti-TB treatment, history of anti-TB adverse reactions, history of liver diseases, HBSAg and pattern of taking anti-TB drugs between the two groups. 75 patients (67.6%) in scheduled monitoring group and 94 (58.0%) in passive detection group took preventive liver protective drugs during the anti-TB treatment (P = 0.111). 57 (51.4%), 3 (2.7%), 5 (4.5%) and 10 (9.0%) patients in scheduled monitoring group took finished manufactured herbal preparations, combinations of vitamins and other non-herbal substances, pharmaceutical preparations and combinations of above liver protective drugs, respectively. On the contrary, 59 (36.4%), 5 (3.1%), 7 (4.3%) and 23 (14.2%) patients in passive detection group were prescribed four categories of liver protective drugs above, respectively.

**Table 1:** Baseline characteristics of 273 patients with liver dysfunction in ADACS

**Category**	**Scheduled monitoring group** **(n = 111)**	**Passive detection group** **(n = 162)**	**P value**
Age, median years (IQR)	40(29,53)	40(28,55)	0.243
Male	89(80.2%)	123(75.9%)	0.407
Married	86(77.5%)	130(80.1%)	0.697
Income per year, median¥(IQR)	10000(5000,20000)	10000(5000,15000)	0.647
Weight			
<50 kg	27(24.3%)	44(27.2%)	0.600
≥50 kg	84(75.7%)	118(72.8%)	
Type of anti-TB treatment			
Primary treatment	95(85.6%)	143(88.3%)	0.514
Re-treatment	16(14.4%)	19(11.7%)	
History of anti-TB adverse reactions			
Yes	3(2.7%)	2(1.2%)	0.668
No	108(97.3%)	160(98.8%)	
History of liver diseases^*^			
Yes	7(6.3%)	9(5.6%)	0.795
No	104(93.7%)	153(94.4%)	
HBSAg positive	21(18.9%)	32(19.8%)	0.940
Pattern of taking anti-TB drugs			
Every day	9(8.1%)	17(10.5%)	0.510
Every other day	102(91.9%)	145(89.5%)	
Usage of preventive liver protective drugs			
Finished manufactured herbal preparations	57(51.4%)	59(36.4%)	0.145
Combinations of vitamins & other non-herbal substances	3(2.7%)	5(3.1%)	
Pharmaceutical preparations	5(4.5%)	7(4.3%)	
Combinations of above drugs	10(9.0%)	23(14.2%)	

Note: ^*^Including hepatitis B, icterohepatitis and cholelithiasis.

### Clinical features

91 (33.3%) of 273 patients did not have any clinical symptoms, including 8 with severe hepatotoxicity. Among 111 patients in scheduled monitoring group, only 20 patients (18.0%) developed symptoms of nausea (11 patients, 55%), vomiting (9 patients, 45%), anorexia (5 patients, 25%) and abdominal symptoms (4 patients, 20%) after detecting liver dysfunction. None of the patients developed icterus and dark urine. All 162 patients (100%) in passive detection group were symptomatic. 116 (71.6%), 110 (67.9%), 31 (19.2%) and 51 (31.5%) patients experienced nausea, vomiting, anorexia and abdominal symptoms, respectively. 5 patients (3.1%) developed icterus and 1 patient (0.6%) developed dark urine (Table 2).

**Table 2:** Clinical features of liver dysfunction in the study groups

**Clinical features**	**Scheduled monitoring group (n = 111)**	**Passive detection group (n = 162)**	**P value**
Symptomatic liver dysfunction	20 (18.0%)	162 (100.0%)	<0.001
Liver dysfunction finding time, median, days (IQR)	52 (30, 61)	39 (28, 60)	0.205
Severity of liver dysfunction			
Mild	70 (63.1%)	97 (59.9%)	0.443
Moderate	28 (25.2%)	37 (22.8%)	
Severe	13 (11.7%)	28 (17.3%)	
Requirement of hospitalization	2 (1.8%)	18 (11.1%)	0.004

The median interval in days between the initiation of anti-TB treatment and the development of liver dysfunction was 52 (IQR: 30, 61) for scheduled monitoring group and 39 (IQR: 28, 60) for passive detection group. The difference between patients in the two groups was not statistically significant (P = 0.205) (Table 2).

In terms of the severity of liver dysfunction, more patients (28 patients, 17.3%) in passive detection group experienced severe liver dysfunction in comparison with those in scheduled monitoring group (13 patients, 11.7%), although there was no statistically significant difference between the two groups (P = 0.443). Besides, 8 patients with severe hepatotoxicity in scheduled monitoring group did not have any clinical symptoms. Only 2 patients (1.8%) in scheduled monitoring group required hospitalization, whereas in passive detection group a total of 18 patients (11.1%) were hospitalized. The difference between the two groups was statistically significant (P = 0.004) (Table 2). When analyzing patients with liver dysfunction finding time less than 1 month, no one in scheduled monitoring group required hospitalization in contrast to 8 patients (11.9%) in passive detection group (P = 0.025).

### Prognosis of liver dysfunction and impact on anti-TB treatment

Regarding the prognosis of liver dysfunction, 1 patient in passive detection group was excluded from the analysis due to death of lung cancer. 109 patients (98.2%) recovered, 2 patients (1.8%) continued with abnormal transaminase, and none of the patients died in scheduled monitoring group, whereas in passive detection group 158 patients (98.1%) recovered, 1 patient (0.6%) continued with abnormal transaminase, and 2 patients (1.3%) died of hepatotoxicity. A 68 years old woman who experienced ALT/AST elevation to 693.1/510.2 U/L after 35 days of anti-TB treatment was with no improvement after hospitalization and finally died of hepatic failure on the 45th day; another 69 years old man who experienced ALT elevation to 210 U/L with jaundice after 65 days of anti-TB treatment died after 1 week in hospital. The propensity score weighting analysis showed that there was not significantly different between the two groups (P = 0.965).

In scheduled monitoring group, 39 patients (35.1%) changed their anti-TB treatment, whereas 92 patients (56.8%) in passive detection group changed the treatment. The difference was statistically significant by propensity score weighting analysis (P = 0.001). As shown in Table 3, more patients in passive detection group interrupted and discontinued the anti-TB treatment in comparison with patients in scheduled monitoring group (P = 0.007 and P = 0.026, respectively).

**Table 3:** Impact of liver dysfunction on anti-TB treatment in the study groups

**Changes of anti-TB treatment**	**Scheduled monitoring group (n = 111)**	**Passive detection group (n = 162)**
No change	72 (64.9%)	70 (43.2%)
Interruption	15 (13.5%)	48 (29.6%)
Dose decrease	4 (3.6%)	1 (0.6%)
Drug replacement	6 (5.4%)	11 (6.8%)
Discontinuation	2 (1.8%)	10 (6.2%)
Changes in medical administration	3 (2.7%)	11 (6.8%)
Interruption with drug replacement	7 (6.3%)	9 (5.6%)
Interruption with change in medical administration	1 (0.9%)	1 (0.6%)
Dose decrease with change in medical administration	1 (0.9%)	0 (0.0%)
Change in medical with discontinuation	0 (0.0%)	1 (0.6%)

When analyzing the impact of liver dysfunction on TB condition, 4 patients in passive detection group were excluded because they could not be followed up for TB treatment outcomes due to discontinuation after the onset of hepatotoxicity (3 patients) and death of lung cancer (1 patient). 89 patients (80.2%) in scheduled monitoring group and 112 patients (70.9%) in passive detection group had TB cured on time. None of the patients had TB aggravated or died in scheduled monitoring group, whereas there was 1 (0.6%) with TB aggravation and 2 (1.3%) deaths in passive detection group (Table 4).

**Table 4:** Impact on TB condition in the study groups

**TB condition**	**Scheduled monitoring group (n = 111)**	Passive detection group (n = 158)^*^	**P value^#^**
Cure on time	89 (80.2%)	112 (70.9%)	
Prolongation	22 (19.8%)	43 (27.2%)	0.084
Aggravation	0 (0.0%)	1 (0.6%)	
Death	0 (0.0%)	2 (1.3%)	

Note: ^*^ 4 patients were excluded due to lost to follow up after discontinuation and death of lung cancer.

^#^ P value was calculated after propensity score weighting analysis.

## Discussion

This study was based on a well-established prospective cohort and reflected the effect of routine monitoring in a real-world population with geographically and economically diverse areas in China. However, a major methodological problem in this observational study is that investigators have no control over the intervention assignment, which is likely to result in confounding and biased estimation of intervention effects [19-22]. To account for these differences in observed covariates, propensity scores were applied in this analysis.

Among several propensity score methods, SMRW and matching estimated most closely approached results observed in the clinical trials [23]. In comparison with matching, SMRW has more advantages [24]: 1) data from all patients are used so that variance is more closely to the study population; 2) process for SMRW is much easier than propensity score matching. Based on all above, we used SMRW to balance all available covariates and estimate the effect of routine monitoring in this study.

In the present study, 1/3 of the patients with liver dysfunction did not have any clinical symptoms, including 8 with severe hepatotoxicity, which indicated the important role of routine monitoring in identifying asymptomatic liver injury. Liver injury could be fatal if it was not recognized in time [12], thus routine monitoring could be helpful to detect liver damage early so as to apply appropriate interventions in time and improve the prognosis. Our results showed that there was no death and only 2 patients (1.8%) in hospital in scheduled monitoring group. 35.1% changed their anti-TB treatment. In contrast, in passive detection group 11.1% required hospitalization and 2 patients (1.3%) died. More than half of the patients changed their anti-TB treatment. Therefore, routine liver function monitoring was associated with less hospitalization, better prognosis and better compliance of anti-TB treatment in this study. A study by Agal et al. [25] showed that 21 (10.5%) of 200 TB patients who accepted regular liver function monitoring (every week for the first month, then fortnightly for the next 2 months and then monthly until the end of therapy) developed ATLI and no one died, whereas 16.6% of the patients without periodic monitoring died and 75% developed icteric hepatitis. In a study by Mcneill et al. [26], the rate of severe ATLI in patients receiving RZ prophylaxis for latent tuberculosis infection (LTBI) reduced from 5% to 0% with periodic monitoring. Another study [27] with H prophylaxis for LTBI indicated that no severe ATLI developed on routine monitoring.

Biochemical abnormalities generally occur before clinical symptoms or signs of liver injury develop, thus monitoring of liver function can detect liver injury ahead of the symptomatic period, prevent serious ATLI and avoid incompliance of anti-TB treatment [12,16]. In our study, the median liver dysfunction finding time of passive detection group was 39.0 days, which indicated that monitoring in the first month after the initiation of anti-TB treatment was important. Subgroup analysis for patients with liver dysfunction finding time less than 1 month showed that significantly more patients required hospitalization in passive detection group. More patients in passive detection group changed their anti-TB treatment and were likely to have a poor prognosis of hepatotoxicity, although there was no significant statistically difference between the two groups.

Some limitations need to be noted. First, the ADACS study was not specially designed to evaluate the effect of regular liver function monitoring, only two routine tests (baseline and within 2 months after initiation of anti-TB treatment respectively) were conducted, thus it may limit the ability of detecting liver dysfunction earlier. Secondly, although we controlled a large set of factors that potentially differed between the groups at baseline using propensity score method, there are still some unknown confounders which may not be balanced. Propensity scores are not magic bullets capable of eliminating all the bias of observational studies [28]. Besides, the study did not collect patients’ information on HIV infection and alcohol consumption which are important risk factors of hepatotoxicity. Finally, owing to the only two routine tests in our study, it is still not clear what frequency or intervals between tests may be optimal. And further studies on compliance and cost effectiveness of monitoring are needed.

## Conclusions

In conclusion, scheduled monitoring is effective in identifying asymptomatic liver damage, reducing hospitalization rate and improving compliance of anti-TB treatment. It is likely to alleviate hepatotoxicity and reduce mortality. At least once monitoring should be performed during the first month after initiation of anti-TB treatment, and monitoring in the second month is highly suggested if available.

## Competing interests

The authors declare that they have no competing interests.

## Authors’ contributions

SSW collected and sorted the data, wrote and revised the manuscript. SYZ contributed to the design, acquisition of study data and revised the manuscript critically. YYX, DHT and YPD also contributed to the design of the study. SMC, XMW, YLY, FYL and DYH contributed to the acquisition of study data. XZL, YZ, SWT and ZRY collected and sorted the data.

## Pre-publication history

The pre-publication history for this paper can be accessed here:

http://www.biomedcentral.com/1471-2458/12/454/prepub
